# Herbal Remedies for Skin Diseases in Serbian Folk Medicine: A Review of 19th- and 20th-Century Practices

**DOI:** 10.3390/plants15081246

**Published:** 2026-04-17

**Authors:** Jelena Živković, Katarina Šavikin, Nektarios Aligiannis, Marko Pišev

**Affiliations:** 1 Institute for Medicinal Plants Research “Dr. Josif Pančić”, Tadeuša Košćuška 1, 11000 Belgrade, Serbia; ksavikin@mocbilja.rs; 2 Division of Pharmacognosy and Natural Products Chemistry, Department of Pharmacy, School of Health Sciences, National and Kapodistrian University of Athens, Panepistimiopolis, 15771 Athens, Greece; aligiannis@pharm.uoa.gr; 3 Department of Ethnology and Anthropology, Faculty of Phylosophy, University of Belgrade, Čika Ljubina Street 18-20, 11000 Belgrade, Serbia; marko.pisev@f.bg.ac.rs

**Keywords:** skin, hair problems, bites, herbal remedies, ethnomedicine

## Abstract

This study explores Serbia’s rich ethnopharmacological heritage by systematically documenting the traditional use of medicinal plants for treating skin diseases during the 19th and 20th centuries. Drawing on key ethnographic sources—including monographs, scholarly articles, and field reports—the review analyzes historical records of folk medicine practices and their cultural contexts. A total of 164 plant species from 63 botanical families, as well as one mushroom species, were identified as being used in the treatment of skin-related conditions classified according to the International Classification of Primary Care. Reported ailments were grouped into three main categories: hair and scalp disorders, bites, and various inflammatory skin conditions such as eczema and psoriasis. Remedies for wound healing were the most frequently documented, both in terms of application and diversity of plant species employed. By preserving and systematizing this historical knowledge, the study provides a valuable foundation for future pharmacological and dermatological research, highlighting the continued relevance of traditional remedies in modern clinical practice.

## 1. Introduction

Historical ethnobotanical research has become an increasingly important field of study. By analyzing this data, we can gain deeper insights into the evolution of Local Ecological Knowledge, how societies have utilized local plant species, and how they have interacted with and shaped their environment over time [1]. In Serbia, medicinal plants have been used to treat various health issues since medieval times. The earliest records on this subject date back to the 14th century (*The Hodoch Codex*) and the 16th century (*The Chilandar Medicinal Codex*) [2]. The importance of herbal medicine grew over time, and in the 19th and 20th centuries, the confidence in the power of herbal remedies was so deeply rooted in the traditional worldview that many of the peasants in need of receiving professional medical care underwent hospital treatment only when all other (i.e., customary) methods of healing failed to develop a favorable outcome. This widespread belief did not seem to fade until the post-WWII, socialist period in Serbia, when the institutions of the central state, in both education and health care, succeeded in extending their reach beyond the urban areas.

Hair and skin have often been viewed as intermediaries between the individual and society. In the past, individuals with skin diseases were often informally isolated, as their condition was frequently linked to venereal diseases. The treatment of skin diseases has always been complex and prolonged, especially in the case of chronic and persistent conditions.

The development of dermatology in Serbia began in the 19th century, following the First and Second Serbian Uprisings [3]. At that time, living conditions, particularly sanitary conditions, were extremely poor. The country lacked doctors, hospitals, schools, and infrastructure, and the vast majority of the population was illiterate. Migrations and the expansion of trade relations contributed to the spread of communicable diseases, which left a significant mark on the 19th century. In these circumstances, folk healers played a crucial role. However, not everyone could assume this role, as it was largely determined by gender and age. Older women, especially widows who had ceased menstruating, were particularly favored for this vocation [4]. These aged women were called “čiste žene” (clean women), or “čiste babe” (clean grandmothers), and were considered to be ritually pure, and therefore particularly effective in nullifying the impure or unholy “essence” of the disease in the patient’s organism. Nevertheless, male “empiricists” also could, and indeed often did, serve as folk healers, especially when their practice was limited to treatment of open and bloody wounds, as well as injuries caused by firearms or knives [5,6]. Some of them possessed the official permits issued by the authorities for their practice [6]. The situation improved toward the end of the 19th century when the first hospitals were built in the country and the number of trade doctors reached a few hundred. However, the vast majority of the population was still poorly educated. During the two World Wars, the health care system was overburdened and largely destroyed [7]. A large number of doctors and other medical personnel disappeared during these wars. Recent research indicates that ethnomedicinal knowledge was deeply rooted in the lifestyle of rural populations engaged in agriculture, animal husbandry, and other nature-dependent activities, which required an intimate understanding of local resources [8]. Despite significant socio-economic transformations that have disrupted traditional practices and contributed to knowledge loss, elements of this heritage have persisted, underscoring the importance of its documentation and preservation.

This paper aims to explore plant-based treatments used in Serbian folk medicine for skin disorders during the 19th and early to mid-20th centuries. This study excludes rituals, spells, and magical practices associated with traditional healing, as the analysis of such customs, while enlightening, does not directly align with the main objectives of this paper. We have chosen to focus on this material because the texts analyzed provide valuable insights into cultural knowledge that is now partially or entirely forgotten, abandoned, or transformed.

## 2. Results and Discussion

Results of our study are shown in Table 1, where the botanical names of the plant species are organized in alphabetical order together with their local names, part(s) used, mode of preparation, and application. This study identified 164 plant species from 63 families, as well as one mushroom species, all of which are used in the treatment of various ailments classified as skin diseases according to the International Classification of Primary Care. After comparison with contemporary ethnobotanical investigations conducted on the territory of Serbia [2,8,9], the authors have observed that a significantly lower number of plant species was recorded in modern studies. Frequently used families were: Asteraceae (14 taxa), Lamiaceae (13 taxa), Solanaceae (10 taxa) and Rosaceae (8 taxa). Results from the previous studies [2,9,10] also confirmed that plant species from the families Asteraceae and Lamiaceae are predominantly used in Serbian traditional medicine. These families include cosmopolitan plant species that are readily accessible in the ecosystems of the investigated area. Therefore, their widespread use in traditional medicine can partly be attributed to their abundance in the Balkan flora. Interestingly, in contrast to contemporary ethnobotanical research, our study recorded a high usage of plants from the Solanaceae family. These plants were primarily used to treat wounds, skin pustules, carbuncles, furuncles, psoriasis, lichen, and oedema. The observed decline in their use today is likely due to their toxicity [11]. Solanaceae species are notably rich in alkaloids, primarily tropanes, whose toxicity to humans ranges from mildly irritating to lethal, even in small quantities. However, when used in appropriate dosages, these plants can serve as valuable therapeutic agents, functioning as antimicrobials, insecticides, and anti-infective agents. In addition to alkaloids, members of the Solanaceae family are reported to contain a variety of bioactive secondary metabolites, including flavonoids, lactones, lignans, steroids, simple phenols, sugars, terpenoids, and antimicrobial peptides [12]. Considering this, investigating the efficacy of plant species from the Solanaceae family in treating skin disorders could hold potential for future research.

Among the plant parts, leaves were the most commonly used (21%), followed by aerial parts (17%), roots (16%), fruits (11%), and seeds (9%). A possible reason for this could be the ease of collecting leaves and aerial parts, especially in situations that require a quick response, such as treating bites, burns, or cuts. Most plant species and their products were applied externally (60.98%). A smaller proportion (9.15%) was used both internally and externally, while 4.27% was only used internally. The data also indicated that herbs were processed and consumed in various ways, both fresh and dried. When fresh, the plant parts were either applied directly or mashed and mixed with water, milk, vinegar, oil, or other substances. In paste form, the plant parts were typically combined with ghee, honey, lard, or butter before application. For plants like *Arnica montana*, *Fumaria officinalis*, *Galium verum*, and *Conium maculatum*, juice was squeezed from the fresh parts and used. This method of preparation is supported by studies indicating that fresh plant juice has a stronger antimicrobial effect compared to alcoholic extracts [13,14]. Dried plants were primarily soaked in brandy (‘rakija’) or water for a few days to create an infusion or decoction. Additionally, the powder obtained from dried plant parts was sprinkled directly onto the affected skin areas. Another characteristic of traditional medicine in the 19th and early 20th centuries was the combination of plant species with organic materials to enhance their effects. For example, yarrow was mixed with urine and applied to the skin, while wounds treated with a corn flour poultice were rinsed with urine. Ha et al. [15], demonstrated that the application of urine significantly reduced the period of epithelization in experimentally induced wounds in rats. At the same time, collagen synthesis was stimulated, likely due to the presence of urea and growth factors in the urine. Inorganic compounds were also used to enhance the action of natural compounds. According to our findings, gunpowder was used in traditional recipes alongside garlic or tobacco. The reason for this usage lies in the presence of saltpeter, a component of gunpowder. Saltpeter has antibacterial properties due to the release of nitrites, which are then converted into nitric oxide [16]. In some cases, for the preparation of traditional herbal products, it was also important how the plant species were collected. For example, according to Milosavljevic [17], *Hieracium pillosela* should be harvested in silence, before the sun, to be effective in lichen treatment. Also, reported medications were mainly prepared from one plant, but for some of them, more than one plant species was recorded. Mainly, they were used in the treatment of one to three ailments, while *Nasturtium officinale*, soaked in water, was used as a panacea.

**Table 1:** Plant species and their traditional uses for the treatment of skin diseases and conditions in Serbia during the 19th and 20th centuries.

Scientific Name, [Old Latin Name], Vernacular Serbian Name, Family Name	Part(s) Used	Medical Use (Preparation Form)	References
*Achillea millefolium* L., hajdučka trava, hajdučica, stoliska, vučja trava, sporiš, kučja trava, mekuška, Asteraceae	Aerial part	E: wounds (balm)	[18]
		E: contusion (when somebody hurts their finger, the plant should be baked, mashed, and applied to the injured place. Also, plant could be mixed with brandy ’rakia’)	[19]
		E: wounds (fresh plant should be squeezed and mixed with urine applied on skin)	[20]
		E: cuts (egg white cooked in vinegar should be applied first, and after that, fresh *A. millefolium* should be applied directly)	[4]
		E: wounds (mashed and applied directly)	[21]
	Leaf	E: bleeding wounds (applied in combination with plantain)	[22]
*Actaea spicata* L., otrovnica, beli gorščanik, Ranunculaceae	Aerial part	E: scabies, head lice, and nits	[23]
*Aconitum vulparia* Rchb., mlječara, Ranunculaceae	/	E: for neglected wounds filled with maggots. The plant should be dried and pulverized, so the wound should be covered with this powder	[22]
*Aegopodium podagraria* L., aptovka, jarčevac, sedmolist, Apiaceae	/	E: wounds	[22]
*Allium cepa* L., crni luk, Amaryllidaceae	Bulb	I: snakebites (as antidote)	[18]
		E: furuncle, burns (directly applied)	
		E: finger contusion	[24]
		E: skin ulcer. Soup should be scraped on the roasted onion, sprayed with oil, and subsequently placed over the ulcer	[25]
		E: for ulcer breakout. After the onion, plantain should be attached to the skin	[26]
		E: contusions. Crushed onion should be attached to the bruised tissue	[26]
		E: contusions. Finely crushed and salted onion should be attached to the bruised tissue without wounds	[19]
		E: contusions. Halved and salted onions should be placed on the bruised skin	[21]
		E: against panaritium. Onion fried in oil should be applied lukewarm on the affected skin	[22]
	Scale leaves	E: dandruff. The head should be washed 3 to 9 times with water in which onion scale leaves have been cooked	[4]
	Bulb	E: dandruff. A large quantity of onions should be crushed and cooked in water. Subsequently, this water should be used for rinsing the head	
		E: For ulcer. Baked onion and live coal sprayed with petroleum should be placed on the ulcer so that the wound festers as soon as possible. Also, a baked onion and scraped soap should be placed on the ulcer in order to cause it to fester and then burst	[19]
*Allium ampeloprasum* L., vinogradarski luk, divlji luk, ljutika, Amaryllidaceae	/	E: treatment of contusions, skin ulcers, and purulent pimples	[27]
	Leaf	E: against insect stings. Leaves should be sprinkled with warm ash, oil, and attached to the affected area	[17]
*Allium sativum* L., beli luk, Amaryllidaceae	Bulb	E: tinea, scabies, furuncle, wounds (traditional balm), dog bite	[18]
		E: snakebites (it is placed on the wound over the top of the cabbage leaf)	
		E: against pimples. A garlic bulb should be crushed together with gunpowder (in a quantity sufficient for one cartridge). A cake should be made from this mixture, large enough to cover the pimple. Afterwards, a small quantity of brown sugar should be smashed with ammonium chloride and used to cover the cake. This cake should stand on the affected area for 24 h	[28]
		E: against pain in the finger. An egg should be taken, and a mixture of garlic, brandy (called ‘rakija’) and chicken excrement should be made within. The finger should be kept in it for 24 h	[24]
		E: When the nail falls off the finger. It should be covered with crushed garlic mixed with chicken excrement	
		E: lichen planus. Chewed garlic should be applied to affected skin every morning	[24]
		E: against boils. Crushed fresh garlic mixed with oil and vinegar should be applied directly to boils	[4]
		E: against cutaneous antrax. Garlic, gunpowder and sulfur should be mixed in a mortar and placed with a bandage on the skin for at least one day and one night	[17]
		E: snakebite. Crushed garlic should be applied to the area together with gunpowder	[17]
		E: for the “chicken pimple”. Related to small children: purulence appears on the leg, and black blood can be seen there. As a medication, apart from garlic, last year’s bacon is also placed on the infected spot, meaning the bacon made during the last year; chicken excrement is added	[27]
		E: for hair-strengthening treatment. Chopped garlic is mixed with brandy (komovica), castor oil and live egg. This “packaging” should be washed with infusions of laurel, rosemary, sage, or walnut leaves and komovica (brandy)	[29]
*Althaea officinalis* L., beli slez, belodun, pitomi slez, Malvaceae	Root	E: carbuncle, wounds	[18]
		E: from sugreb (skin rash, manifested in the form of small pimples or sklopac. It causes itching. People across the former Yugoslavia believed that someone catches sugreb when he/she steps on a place where wolves or dogs previously scratched their nails). To treat it, it is necessary “to find a root of a marshmallow, then to crush it down and sunk it into the brandy for 24 h. After that, the root is trashed away while the brandy is used for rubbing the infected spots”	[30]
		E: for treatment of wolves’ sugreb (rash). In the region of the Homolje mountains, there was a belief that the sugreb (rash) was manifested in tiny white measles which became red after screeching, and from which the older children usually suffer, as they are the ones who like to wander around the dirty places. If they step onto the wolves’ sugreb (the place which was dug out by the wolf’s front legs and afterwards which he covered with earth), the villagers from the Homolje villages believed that the following rash could be lethal. This is why they would find the root of marshmallow, to crush it down and then to melt it into the brandy, where it will be submerged for 24 h. Afterwards the root is thrown away and that brandy is used for rubbing the infected area three times	[17]
		E: against skin ulcers (called “nicina”). Root should be boiled in milk until porridge is made which can be applied directly on skin	[28]
		E: against skin blisters and bumps. Baked marshmallow, mixed with ammonium chloride, should be placed on the affected skin	[4]
		E: wounds (root is boiled in water, and this water is further used for wound rinsing)	[21]
*Amaranthus cruentus* L., crveni štir, trator, Amaranthaceae	/	E/I: erysipelas	[31]
*Aquilegia vulgaris* L., [A. cornuta Gilib.], kandilica, kandilka, zvonce, popova kapa, Ranunculaceae		E: scabies	[23]
*Aristolochia clematitis* L., vučja jabuka, popova muda, kokotinja, Aristolochiaceae	Root	E: For chronic wounds. Root should be washed seven times and then well dried. Root powder should be mixed with honey and applied as a poultice	[32]
*Armoracia rusticana* P.Gaertn., B.Mey. & Scherb., [*A. lapathifolia* Gilib.], ren, hren, morska rotkva, Brassicaceae	Root	E: against the disease called “poganica”. This disease has been considered by people in Herzegovina to be the result of contusion. It develops when the blood cannot circulate due to the trauma, so that it becomes ‘bad’. The root of the horseradish should be crushed and applied on the area	[5]
		E: contusion. Grind root should be mixed with flour, and then the mixture should be applied to the injured area directly	[33]
		E: contusion (mashed root applied after mixing with vinegar and oil)	[4]
*Arctium lappa* L., [*Lappa major* Gaert.], repušina, čičak, Asteraceae	Root	E: absorbing pus from wounds (mashed root applied as a wound dressing)	[29]
		E: wounds	[22]
		E: skin carbuncle (spissum extract prepared with fresh root)	[23]
		E: against facial dandruff, “rusa”, lichen with crusts, eczema, and pimples—the middle of the fresh root or thick water extract (decoction) is used	
*Arnica montana* L., trava od mojasila, brđanka, moravka, tičji neven, Asteraceae	/	E: chronic eczema (plant should be crushed and juice should be squeezed from the plant. Subsequently juice should be applied directly on the affected skin)	[5]
*Avena sativa* L., zob, ovas, siljevina, Poaceae	Seed	E: against “erysipelas”. Embers should be heated, and two seeds of corn and oat should be pressed on it with an axe. The fatty substance that comes out should be applied on the skin	[25]
*Beta vulgaris* L., cvekla, blitva, pasja blitva, Amaranthaceae	Fruit	E: erysipelas (cut into pieces and applied directly on damaged skin)	[34]
*Brassica oleracea* L., broskva, kupus, Brassicaceae	Seed	E: “rusa” (direct application of the pulverised seed)	[18]
	Leaf	E: infected wounds, burns, erysipelas (leaf directly on damaged skin)	
	Leaf	E: erysipelas (fresh leaf applied directly on the skin)	[34]
	Leaf	E: burns (fresh leaf applied directly on the skin)	[17]
	/	E: burns. Sauerkraut juice should be mixed with egg white and salted and then applied on the burned skin	[4]
*Buxus sempervirens* L., šimšir, zelenika, Buxaceae	Leaf	E: prevents hair loss (combined with fresh nettle leaves and leaves and seeds of jewels, a tincture should be prepared and then rubbed on the scalp skin)	[23]
*Calendula officinalis* L., neven, Asteraceae	Aerial part	E: juice squeezed from the aerial part should be used for compress preparation and applied on pus-filled pockets in the superficial layer of the skin with yellow crust	[23]
*Cannabis sativa* L., konoplja, Cannabaceae	Seed	I: subcutaneous abscess (decoction)	[18]
		E: against crusts (in children). When a child has crusts on their head, hemp seeds should be crushed in a wooden jar, mixed with fine oil and applied directly on the crusts	[17]
	Leaf	I: against skin rash, skin lichen, eczema, skin tuberculosis, pityriasis, impetigo	[18]
*Capsicum annuum* L., paprika, Solanaceae	Fruit	E: carbuncle (applied directly)	[18]
		E: skin pustules (ripe peppers should be boiled, and stuck to the pustule. Peper should stay on the skin for 24 h)	[17]
		E: against hair loss	[23]
*Chelidonium majus* L., rusopas, rusa, Papaveraceae	Latex	E: furuncles on the leg, warts	[18]
	/	E: against crusts. Well crushed, the plant should be mixed with salt, oil and ‘rakia’, and this balm should be applied to crusts	[27]
*Citrus aurantium* L., narandža, Rutaceae	Fruit	E: pruritus (boiled with salt)	[18]
*Citrus limon* (L.) Osbeck, limun, Rutaceae	Fruit	E: skin blister (lemon slice applied directly)	[24]
*Coffea arabica* L., kafa, Rubiaceae	Seed	E: burns, rash (roasted coffee directly on affected skin)	[18]
		E: small burns (salve the skin with fat, cover it with ground coffee and subsequently bandage)	[19]
*Conium maculatum* L., kukuta, Apiaceae	/	E: bee bite (juice from fresh pressed plant mixed with vinegar, oil and benzine applied on the bite spot)	[4]
*Cornus mas* L., dren, drenjina, Cornaceae	Fruit	E: skin efflorescence	[18]
*Corylus avellana* L., leska, Corylaceae	Seed	E: scabs (“in Bosanska Krajina the one who has scabs uses hazelnut ointment on his face and hands”)	[18]
	Seed coat	E: against skin bumps. In the hazelnut seed coat, a mixture of copper, sulphur, small amount of gunpowder should be prepared with saliva. This mixture should be placed on a bump and after 24 h “it has to pass”	[21]
*Crataegus oxyacantha* L., glog, Rosaceae	Fruit	E: carbuncle (fruit pulverised on the moving rock is the integral part of the balm)	[18]
*Cucucmis sativus* L., krastavac, Cucurbitaceae	Fruit	E: against “nicina” (swelling of the skin as a result of lymph gland inflammation). Baked *C. sativus* fruit should be mashed and applied on the skin	[4]
	Seed	E: seeds should be mashed and mixed with water. It should be applied as a dressing against ulcers and abscesses)	[23]
*Cucurbita lagenaria* L., tikva, Cucurbitaceae	Fruit pulp	E: burns (compress)	[18]
*Cucurbita pepo* L., bundeva, “bela tikva”, bundevka, beli dulek, Cucurbitaceae	Fruit	I: psoriasis (“sick person every day eat pumpkin roasted or boiled, salted or sweetened, prepared in different ways, until the patient had it enough”)	[35]
		E: oedema (cooked or baked, mixed with sugar and applied directly)	[19]
		E: against burns. Pulp with seeds should be soaked in milk and applied on affected area	[4]
*Cyclamen purpurascens* Mill. [*C. europaeum*], skrež, skrž, šumska ciklama, Primulaceae	Whole plant	E: wounds. Crushed plant should be applied on the wound	[36]
*Dysphania ambrosioides* (L.) Mosyakin & Clemants, Amaranthaceae	Leaf	E: wounds (mashed leaf soaked in hot water)	[22]
*Daucus carota* L., šargarepa, mrkva, žuta repa, Apiaceae	Root	I: juice obtained from the fresh carrot should be taken orally against contagious crusts in kids with impetigo	[23]
*Datura stramonium* L., tatula, bazdulja, trava od zlikovca, kužnjak, Solanaceae	Leaf	E: old wounds (washed in good “rakia” and applied on affected area)	[35]
	Leaf and root	E: against two inflammation types on skin—*Panaritium* i *Phlegmona.* Leaf should be directly applied on the skin, while root should be first dried and crushed and as a pulvis applied over the wounded area	[34]
	/	E: against oedema. Crushed plant soaked in strong brandy should be applied on the oedema 2–3 times per day	[24]
			[19]
*Delphinium consolida* L., [*Consolida regalis* Gray], žavornjak, čelebin-perčin, Ranunculaceae	Aerial part	E: against scabies, nits	[23]
*Dianthus caryophyllus* L., karanfil, garofilje, Caryophyllaceae	Aerial part	E: scalp scabs (plant powder mixed with milk)	[18]
*Dictamnus albus* L., jasenak, Rutaceae	Aerial part	E: wounds (balm)	[18]
*Dipsacus fullonum* L., trava ustava ili češljuga, Caprifoliaceae	/	E: against hair loss	[4]
*Drosera rotundifolia* L., rosulja, Droseraceae	Aerial part	E. warts (juice from the fresh plant directly onto warts)	[35]
*Elaeagnus angustifolia* L., maslenka, mirišljava vrba, dafina, Eleagnaceae	Fruit	E: scabs, scabies (ointment)	[18]
*Elymus repens* (L.) Gould, pirevina, troskot, Poaceae	/	E: used against scubs	[31]
*Eupatorium cannabinum* L., trava “uštuk”, Asteraceae		E: against wounds called “nazdravice” (they appear suddenly on healthy skin by themselves)	[27]
*Euphorbia cyparissias* L., mlečika, Euphorbiaceae	Latex	E: bites (“protects from the bite of green lizard, the wound that hardly anything can help”)	[18]
		E: for skin inflammation that affects the face. Milky sap from the fresh plant should be applied directly on inflamed area	[29]
*Ficus carica* L., smokva, Moraceae	Fruit	E: scorpion stings (juice from fresh fruits applied directly)	[18]
		E: scabs, wounds	
*Frangula alnus* Mill., [*Rhamnus frangula* L.], smrdljika, pasjakovina. krušina, Rhamnaceae	Bark	E: Against itching. Bark should be cooked in water and left overnight. The next day (before the sun), it should be used for bathing (in Herzegovina)	[5]
*Fraxinus excelsior* L., beli jasen, Oleaceae	/	E: anthrax, warts	[18]
*Fumaria officinalis* L., dimnjičarka, rosopas, Papaveraceae	Aerial part	I: eczema (tea or syrup prepared from fresh-pressed plant juice)	[23]
	/	E: scabies (juice from the fresh plant directly applied on infected skin)	[22]
*Galium verum* L., ivanjsko cveće, broćac, Rubiaceae	Aerial part	E: cuts (juice pressed from fresh plant applied directly)	[18]
*Glechoma hederacea* L., trava “samobajka”, dobričica, Lamiaceae	Leaf	E: acne. The leaf should be smeared with honey and applied to the pimple	[21]
	Whole plant	E: oedema. The plant should be crushed, soaked in strong vinegar, and wrapped on a swollen place	[4]
	Leaf	E: wounds (should be applied as a compress regularly)	[4]
			[22]
	/	E: apply as compression for oedema	[22]
*Hedera helix* L., bršljan, Araliaceae	Leaf,	E: scabs, oedema (boiled leaves as compress), burns, wounds	[18]
	Fruit		
	Whole plant	E: for better hair growth. The hair should be washed with water in which *H. helix* was cooked together with “cord” grass (grass that looks like cord and grows in caves)	[24]
	Leaf	I: against crusts. Leaf should be crushed, mixed with wine and lard, and applied on skin	[32]
*Helianthus annuus* L., suncokret, Asteraceae	Seed oil	I: snakebite. A scorpion should be inserted into the sunflower oil in a glass bottle. It must be a scorpion “that lives under the bark of dry trees and cracks in the rocks, and has a ball at the end of its tail with a spike like a thorn”. When a given scorpion dies in oil, it needs to stand for 40 days. Only then does the medicine become effective, and it can be applied to the bitten area	[21]
*Heliotropium europaeum* L., trava “od vetra”, trava od lišaja, posunac, Boraginaceae	/	E: erysipelas	[34]
*Helleborus odorus* L., kukurek, Ranunculaceae	Root	E: scabies (taking a bath in the water in which the root was immersed)	[18]
		E: scabies, pediculosis (the root boiled with tobacco and false helleborine)	[35]
*Hieracium pilosella* L., zečja loboda, Asteraceae	Aerial part, Root	E: tinea	[35]
	Aerial part	E: wounds (plant should be grounded and applied directly on wound)	[36]
	Whole plant	E: lichen treatment. The plant should be harvested in silence, before the sun and milk released from the plant applied directly on the lichen	[17]
*Hordeum vulgare* L., ječam, Poaceae	Fruit	E: snakebites	[18]
		E: wound (directly applied to wounds after mixing with donkey milk)	
	/	E: scabies. On St. George’s Day, before dawn, the patient should roll naked on young and dewy barley in the field	[21]
	/	E: against crust on the children’s heads. Barley bran should be immersed in water. Crusts should be washed with this water and further smeared with hot olive oil	[5]
*Hylotelephium telephium* (L.) H.Ohba, [*Sedum purpurascens* W.D.J. Koch], kravlje sise, Crassulaceae	Leaf	E: Against skin ulcers and corns. Leaves are thick, and when they should be used, the thin epidermis of the leaf should be peeled off first, and then the leaf is attached to the ulcer or corn only 2–3 times before the disease ends	[26]
*Hyosciamus niger* L., bunika, Solanaceae	Seed	E. wounds. Dried seeds are powdered and used for wound sprinkling	[22]
*Hypericum perforatum* L., kantarion, bogorodičina trava, Hypericaceae	Aerial parts	E: cuts	[18]
	Flower	E: treatment of cuts and wounds. Flower should be soaked in oil in a glass bottle for 40 days.	[21]
			[4]
	Whole plant	E: dog bite. A poultice of smashed plant soaked in oil should be applied on the bitten place	[19]
		E: cuts. The smashed plant should be applied directly as a poultice	
	Whole plant	E: treatment of cuts. Plant should be soaked in oil and heated in the sun for six weeks to be effective	[17]
	Leaf	E: for wounds called “nazdravice”, especially on the finger. Leaf should be crushed, mixed, and water should be further used to pour on the affected area	[18]
		E: for treatment of diseased mammary glands	
*Hypericum rumeliacum* Boiss., trava izdatljivica, Hypericaceae	Flower	E: cuts. Only the yellow flower should be collected from the plant and placed in a glass bottle with oil, where it should stand in the sun for 40 days. The obtained oil is applied directly on the affected area	[28]
*Ilex aquifolium* L., zelenika, Aquifoliaceae	Leaf	E: against “live” wounds. Find a leaf from the plant that grows in the mountain, dry it, pound it finely and spread that powder directly on the wound	[37]
*Inula helenium* L, oman, Asteraceae	Root	E: scabs, pruritic	[18]
		E: hair growth stimulator (washing with the water in which the root was immersed)	
	Whole plant	E: against scabies. The plant should be put in heated water. It is necessary to put a spoon of pig’s fat into the pot, as well as a spoon of salt and a pinch of sulphur. All this is cooked until it “becomes as thick as aspics”, only then is it ready. The treatment itself is performed in the following way: You take a bath in the hot lye where a little salt has been added and some of the spicy soap. You should rub yourself intensely with this lye. Only after that, on the dry skin, the medication is to be placed. This should be repeated each morning until the crust is completely gone	[34]
	Root	E: “against evil crust “. The root of the plant should be crushed and applied “hot” in the evening and left to stay until the morning	[33]
	Root	I: against itching. Roots should be well washed and boiled with water. This water should be taken orally in small doses in the evening	[32]
*Iris germanica* L., perunika, Iridaceae	Root	E: against oedema. Root should be boiled with milk	[22]
*Juglans regia* L., orah, Juglandaceae	Leaf	E: against hair loss. Hair should be washed with water in which walnut leaves were cooked	[24]
		E: against crusts. The patient should take a bath in water in which walnut leaves were boiled. Bathing should be repeated 5–6 times; this was considered a safe remedy. Some patients just soaked the walnut leaves in water, and when the water almost turns black, they take a bath in it	[26]
		E: against smelly sweat. Bath should be made with the water in which walnut leaves have been cooked	[17]
	Fruit	E: against chapped lips. Walnut fruit should be baked, and the remaining charcoal should be used to smear the lips	[4]
*Juniperus oxycedrus* L., crvena venja, crvena smreka, Cupressaceae	Root	E: eczema, psoriasis, scabies	[35]
	Bark	E: wounds (decoction for rinsing)	
*Juniperus sabina* L., [*Sabina officinalis* L.], glušac, somina, Cupressaceae	Top of a young twig	E: warts (balm)	[35]
*Kitaibelia vitifolia* Willd., kadivka, Malvaceae	Leaf	E: against wounds. Leaf should be well crushed and soaked in hot water	[22]
*Lamium album* L., mrtva kopriva, Lamiaceae	Root	E: wounds (the pulverised root or fried on the fat applied directly)	[18]
*Laurus nobilis* L., lovor, lovorika, Lauraceae	Leaf	E: erysipelas	[18]
*Levisticum officinale* W.D.J., [*Angelica Levisticum* All.] Koch, selen, Apiaceae	Root	E: bites	[18]
		E: hair growth stimulation (“the woman grease hears with the oil in which the root was soaked in the evening before St. George’s Day, or wash their heir using water where the root was immersed together with black poplar leaves during the night”)	
*Lilium candidum* L., krin, ljiljan, aleluja, zambak, Liliaceae	Root, leaf	E: against oedema. Roots and leaves should be mashed together in order to obtain poultice	[26]
*Linum usitatissimum* L., lan, ćeten, Linaceae	Seed	E: burns, wounds (flax porridge poultice)	[18]
		E: ingrown toenails (flax porridge poultice)	[35]
		E: when the nail is close to falling off, a dish called “cicvara” should be prepared from flaxseed flour, and it should be placed on the diseased finger	[19]
		E: against skin ulcer (called “nicina”). Seed should be boiled in milk until a thick mass is formed. This mass should be placed on a clean linen cloth and bandaged on the wound	[4]
		E: against skin ulcer (called “nicina”). Seed should be fried in oil and applied on the affected skin	[21]
		E: against carbuncles. Flaxseed should be cooked in milk and applied hot on the place which hurt	[5]
*Lycoperdon perlatum* Pers., [*Bovista gigantea* Quel.] puhara, puša, Agaricaceae	/	E: cuts (applied directly)	[21]
*Lycopodium clavatum* L., prečica, prašuljica, Lycopodiaceae	Powdered dry plant	E: powder for sprinkling on moist and inflamed areas on the skin, especially in small children	
*Lythrum salicaria* L., potočnjak, vrbičica, Lythraceae		E: in eczema, redness. Against skin inflammation (mainly in obese people) and diaper rash	[23]
*Malus domestica* (Suckow) Borkh., [*M. pumila* Mill.], jabuka, Rosaceae	Fruit	E: burns	[18]
		E: bruises (apple vinegar)	
		E: warts (rubbing with sour apple)	[19]
		E: against warts. Warts should be rubbed with apple fruit, after which the apple is buried. It was believed that the warts would withdraw when the apple putrefies	[38]
*Mentha pulegium* L., [*Pulegium vulgare* Mill.], metvica, Lamiaceae	Aerial part	E: erysipelas (pulverised herb mixed with barley flour)	[18]
*Myristica fragrans* Houtt., [*M. moschata* Thunb.], oraščić, Myristicaceae	Seed	E: baldness (greasing the head with the balm obtained from pulverised plant)	[18]
*Nasturtium officinale* R.Br., [*Cardamine fontana* Lam.], trava “ugas”, paskvica, krstovnik, Brassicaceae	/	E: burns. The plant should be crushed and directly applied	[21]
	Leaf	E: wounds (fresh leaf as a poultice)	[4]
	/	I: Panacea. Soaked in water, it should be taken orally to cure all diseases	[4]
*Nicotiana tabacum* L., duvan, Solanaceae	Leaf	E: scabs, cuts (pulverised leaves applied directly), wounds, pruritus	[18]
			[28]
	Leaf	I: ’live’ wounds. It should be smoked regularly	[27]
	Leaf	E: For treatment of bleeding wounds. In addition to tobacco, some gar is placed on the bloody wound, as well as pounded ember and a cloth soaked in petrol	[27]
	/	E: snakebite (leaves were mixed with milk and applied as a paste)	[19]
	Leaf	E: Against scabies. Tobacco should be cooked in the water. The infected person should be rubbed with it. At the end the mixture of gunpowder, gar, sulphur, and bacon is made. All this is melted and afterwards applied on the skin	[39]
	/	E: Against the lichen. It should be covered with tobacco gar	[21]
*Ocimum basilicum* L., bosiljak, Lamiaceae	Aerial part	E: snakebites (juice from fresh plant applied directly)	[18]
		E: wasp or bee sting (fresh plant as a poultice)	
*Onopordum acanthium* L., [*Onopordon acanthium* L.], repušina, Asteraceae	/	E: wounds (applied directly)	[22]
*Papaver somniferum* L., [*P. amplexicaule* Stokes], mak, Papaveraceae	Flower	E: eczema (boiled in water)	[18]
*Pentanema squarrosum* (L.) D. Gut. Larr., [*Inula conyza* (Griess.) DC.], ocrnja, bušina, Asteraceae	Whole plant	E: against contusions. The whole plant should be mixed with chicken excrement, crushed garlic (best if chewed), and red pepper. This mixture should be placed on the finger and afterwards poured with brandy. This compress should stay for 24 h after which the procedure should be repeated	[25]
*Petasites hybridus* (L.) G.Gaertn., B.Mey. & Scherb., [*P. officinalis* Moench.], lopuh, repuv, Asteraceae		E: carbuncle, burns (poultice from plant mixed with soured milk)	[18]
	/	E: wounds (applied directly)	[22]
*Phaseolus vulgaris* L., pasulj-ležak, Fabaceae	Seed	E: against chronic wounds. The seed should be crushed in a mortar and dispersed over the wound	[34]
		E: for dog bite treatment. Halved seed is applied to the skin	[21]
		E: against dog bite. Baked yellow bean should be applied directly to the wound	[19]
		E: against dog bites. A few whiskers from the dog that caused the injury and three grains of dried beans should be placed on the wound	[24]
		E: against burns. Baked and halved yellow bean seeds should be applied to the wound	[4]
		E: against dog bites. Five halved grains of white beans should be placed on the bitten area. Also, powder from the same bean should be sprinkled over the wound. The wound should be smeared with dog fat	[30]
*Pinus sylvestris* L., beli bor, Pinaceae	/	E: wounds (balm)	[18]
	/	E: for all kinds of wounds. A slice of unsalted bacon and one leaf of ivy should be put on a thin pine tree and set on fire to burn. The liquid that falls from the bacon and pine resin is caught in a vessel, and wounds should be treated with it	[5]
	Resin	E: against contusions (melted pine resin should be applied)	[4]
*Pistacia terebinthus* L., smrka, smrdljika, Anacaridaceae	Resin	E: against contusions. *P. terebinthus* resin, frankincense, and sea salt should be mixed in the same proportions, and this mixture should be melted over fire, greased on thick paper, and put on the injured place	[5]
*Plantago major* L., ženska bokvica, Plantaginaceae	Aerial parts	E: wounds	[18]
	Whole plant	E: for treatment of bites. Soaked in oil and applied as poultice	[17]
		E: for extracting puss out of a skin ulcer. The plant should be applied directly	[24]
*Plantago media* L., ocrnja, srednja bokvica, Plantaginaceae	Whole plant	E: against pain in the finger	[25]
*Plantago lanceolata* L., muška bokvica, Plantaginaceae	Leaf	E: against wounds. Fresh leaf should be applied directly	[21]
		E: against wounds. Bandage regularly	[4]
*Polygonatum multiflorum* (L.) All., [*Convallaria odorata* Mill.], pokosnica, saradžika, trava od saradže, Asparagaceae	Leaf	E: treatment of chronic wounds. The leaf should be stuck on the affected area	[28]
	/	E: against the loss of hair. The plant should be boiled in water. Simultaneously, the pitch should be washed in water nine times, and afterwards cooked until it is condensed. This mixture should be used for greasing the head	[28]
*Polygonatum odoratum* (Mill.) Druce, pokosnica, Asparagaceae	Root	E: furuncle, bruises, infected wounds (fresh-grounded rhizome applied directly)	[35]
*Polypodium vulgare* L., slatka paprat, Polypodiaceae	Aerial part	E: snakebites (rubbing the wound with the plant)	[18]
*Populus tremula* L., [*P. australis* Ten.], jasika, trepetljika, Salicaceae	---	E: snakebites (rubbing the wound with the plant)	[18]
*Portulaca oleracea* L., [*P. officinarium* Crantz], ledenjak, tušt, Portulacaceae	Whole plant	E: for an injured finger. The plant should be crushed and poured with brandy. This should be mixed with a small amount of oxen dung, crushed garlic, and hot paprika and applied to the finger	[25]
*Potentilla erecta* (L.) Raeusch., [*Fragaria tormentilla* Crantz], trava petoprsta, Rosaceae	/	E: against ’live’ wounds (they differ from ordinary wounds since they last for several years, and people believe that something got in a wound and eat it)	[4]
		*P. erecta* should be crushed together with *Lamium album* and poured with brandy (“komovica”) and applied to the wound	
*Primula veris* L., [*P. officinalis* Hill.], jaglika, Primulaceae	Leaf	E: furuncle (compress)	[18]
*Prunella vulgaris* L., [*Brunella officinalis* Crantz], ocrnja, crnj, crnjevac, krštenica, Lamiaceae	Whole plant	E: when the hand hurts. It should be burned, crushed, and sprinkled on the injured place previously smeared with lard	[25]
	/	E: against pustules and pimples	[22]
*Prunus avium* L., trešnja, Rosaceae	Leaf	E: hair loss prevention	[18]
*Prunus cerasus* L., višnja, Rosaceae	Leaf	E: hair loss (hair rinsing with water in which leaves of violet, cherry, sour cherry, and strawberry were boiled)	[18]
*Prunus domestica* L., šljiva, madžarka, Rosaceae	Fruit	E: hornet bite. Vinegar should be prepared from plum fruit, in which cloth should be soaked, coated with honey, and applied to the bitten area	[17]
	Leaf	E: against swelling. “Place a boiled marshmallow or nettle on the edema in order to provoke festering as soon as possible”. Instead of the nettle, it is possible to place the green leaves of the plum, which can reverse the swelling	[24]
	Fruit	E: against the disease called “rusa”. It is characterized by the yellow crusts, which release water, and when it falls, it provokes the new crust.” The dried plum should be used; it should be cut in half and sprayed from above with a tiny amount of ammonium chloride and then placed on the crust	[28]
	Fruit	E: for the treatment of skin ulcer. Dried plum should be salted with ammonium chloride and applied on skin	[4]
	Leaf (in combination with *Fraxinus excelsior* leaves)	E: against the disease called “poganica”, which is characterized by purulent inflammation (when it is on the hand or the leg). Leaves are placed to ease the pain	[5]
*Pteridium aquilinum* (L.) Kuhn., [*Eupteris aquilinium* Newman], bujača, bujad, Dennstaedtiaceae	/	E: stimulating hair growth. Juice from *Pteridium aquilinum* should be mixed with juice obtained from vine	[22]
*Punica granatum* L., šipak, Lythraceae	Fruit peel	E: wounds (dried and pulverised peel dispersed directly on wound)	[40]
*Quercus pubescens* subsp. *Pubescens* L., [*Q. cerris* L.], cer, Fagaceae	/	E: against crusts. Bark should be roasted and crushed, mixed with oil, and applied directly to the skin	[4]
	/	E: against crusts. Ash from *Q. cerris* should be mixed with small amounts of soot from the chimney and lard. The mixture should be greased on the crusts	[28]
*Quercus robur* L., [*Q. borealis* Simk.], hrast, lužnjak, Fagaceae	Acorn	E: against children’s head crusts. Acorn should be burned, charcoal crushed and mixed with young butter or cheese cream (“kajmak”). This mixture should be greased on crusts	[5]
	Bark	E: against feet sweating. It is necessary to bake the bark and pulverize it. This pulvis should be used for “salting” feet in the evening. The procedure should be repeated for seven days	[4]
	Bark	E: for treatment of burns. Tea from *Q. robur* bark mixed with lime should be used for wound rinsing. Afterwards, it should be covered with lard which small amounts of camphor and red pepper	[4]
		Another way: pounded bark should be mixed with wax and frankincense and applied to the burned spot.	
	Fruit	E: against scabies. The oak’s acorn is baked in ardency until it becomes completely black, then it needs to be smashed, and with this powder, the crusts should be covered. It appears that this medicine is not very efficient	[17]
*Raphanus raphanistrum* L., [*Raphanistrum arvense* Wallr.], rotkva, repnjača, Brassicaceae	Root	E: wounds (cleaning wounds with pressed juice of fresh plant)	[18]
		E: burns, hair loss (grated herb mixed with honey applied as a poultice)	
	Seed	E: fungal skin infections (pulverised seeds mixed with water, topically applied)	[35]
	Fruit	E: against ulcers and purulent inflammations. The radish should be boiled and, as a warm compress, placed directly on the affected area	[27]
*Rosa canina* L., šipak, divlja ruža, Rosaceae	Galls on rose stems made by insects (Cynipides)	E: gall is drenched and placed in vinegar or ethanol, and then this liquid is used for drenching the cotton ball, which is later used for rubbing the places on the head where baldness has appeared	[18]
*Rubus caesisus* L., ostruga, divlja kupina, Rosaceae	Leaf	E: treatment of wounds. The leaf should be simply attached to it	[27]
		E: for ulcer treatment. The leaf should be applied directly to make the ulcer “break in” and to remove the pus	[21]
*Rumex crispus* L., [*Lapathum crispum* Moench] štavalj, Polygonaceae	Root	E: as a compress for oedema	[22]
*Rumex obtusifolius* L., [*Lapathum obtusatum* Montad.] štavalj, konjštak, Polygonaceae	Root	E: as a compress for oedema	[22]
*Rumex patientia* L., [*Lapathum hortense* Moench], srpsko zelje, Polygonaceae	Root	I: chronic skin disorders	[23]
*Rumex sanguineus* L., [*Rumex condylodes* M.B.], štavalj, Polygonaceae	Root	E: as a compress for oedema	[22]
*Rumex scutatus subsp. scutatus* L., kiseljak, Polygonaceae	Root	E: as a compress for oedema	[22]
*Ruta graveolens* L., ruta, sedef, Rutaceae	Aerial part	I: bite by a snake and a venomous spider (as an antidote)	[18]
*Salix alba* L., [*S. aurea* Salisb.], bela vrba, Salicaceae	Bark	E: furuncle (peel ash mixed with butter)	[18]
*Salix purpurea* L., [*S. fissa* Wahlenb], crvena vrba, rakita, Salicaceae	/	I: against contusions. The charcoal obtained after burning of wood should be immersed in water and then drink it	[4]
*Salvia officinalis* L., žalfija, Lamiaceae	Leaf	E: wounds (compress)	[18]
*Sambucus ebulus* L., [*Ebulum humile* Garcke], apta, burjan, Adoxaceae	Root, leaf	E: furuncle, oedema	[18]
		E: bite by snake or scorpion (root or leaves poured with water applied directly)	
	Leaf	E: against skin ulcers, leaf should be applied directly	[28]
*Sambucus nigra* L., zova, Adoxaceae	Leaf, bark	E: wounds (balm)	[18]
	Bark	E: treatment of wounds. It is necessary to take “mezgra” of the elderberry (green layer under bark) and mix it with incense, wax, and oil. These ingredients should be well-cooked. Afterwards, this mixture is filtered so that the elderberry parts are excluded while the rest is kept and guarded in a clean bottle	[27]
		E: the balm used for the treatment of gunshot wounds. It should be prepared from the green layer under the bark, wax, and oil	[24]
		E: treatment of wounds. Bark should be peeled from young elderberry branches until reaching the green parts (“mezgre”), which is then rubbed out and crushed. After that, it should be added to the mixture of wax, goats’ fat, and oil (each ingredient added in the same quantity). This mixture is fried in the pot and afterwards placed in the vessel to cool down and congeal. The balm is then glued on a cloth and applied to the wound	[21]
*Saponaria officinalis* L., [*Lychnis officinalis* Scop.], sapunjača, belonoga, Caryophyllaceae	Leaf	I, E: in obsolete skin diseases (with peeling upper layer, swollen glands, and glandular swellings and scrophula). The leaf should be immediately poured and steamed with boiling water	[23]
*Satureja hortensis* L., [*Clinopodium hortensis* L.], čubar, čubrica, Lamiaceae	Aerial part	E: wounds (balm)	[18]
	Flower	I: against erysipelas. Balm should be made according to the following recipes: One handful of savory flowers should be taken and mixed with one handful of marigold leaves, one handful of *Amaranthus peniculatus* leaves with seeds, ten grams of ‘hum’ (unfamiliar to the authors), ten grams of “ćelermenije” (unfamiliar to the authors), one handful of *Mentha arvensis* leaves and one oka of wine (oka—tur. Okka—unit for measuring weight in Turkey equal to 1.250–1.280 kg). All should be mixed in bottle and patient should drink one wine glass of this liquid on an empty stomach for forty days	[32]
	/	I: against “live” wounds. Four to five L of water should be heated. Savory, basil, and dill should be smeared with honey and dipped in warm water. When it starts to boil, patient should be hoisted over with the head covered with a cloth. The steam should be inhaled until the water cools	[27]
		E: after inhalation, the wound should be smeared with balm prepared from wax, oil, and incense to “rejuvenate” the wound	
	Leaf	E: the place of a bee or wasp sting should be rubbed with leaves	[23]
*Scrophularia nodosa* L., trava od šapa, svinjarka, Scrophulariaceae	Root, aerial part	I, E: as tea and warm compress against scrophulosis swelling and wounds. Also, it can be applied to face freckles	[23]
*Scutellaria hastifolia* L., šišak, Lamiaceae	Whole plant	E: for chronic wounds. The plant should be crushed, mixed with brandy (“rakia”) and cow manure, and applied on the skin	[25]
*Sedum acre* L., zebrica, rebruša, sisača, Crassulaceae	Aerial part	E: warts (juice from fresh plant)	[18]
	Root	E: against rabies and dog bites. The root should be crushed and applied on the wound.	[4]
*Sedum telephium* L., bobovnjak, Crassulaceae	Leaf	E: burns (fresh leaves applied directly on wounds), furuncle (juice from fresh leaves)	[35]
	/	E: as a compress for oedema	[22]
	“pero”	E: against painful heel spur, “perje” stiks when the spur breaks	[22]
*Silybum marianum* (L.) Gaertn., zmijina trava, Asteraceae	Whole plant	E: snakebite. The plant should be crushed and applied to the area of the bite	[24]
*Sinapis arvensis* L., gorušica, Brassicaceae	Seed	E: dandruff, scalp scabs (mixed with parsley and common juniper)	[18]
*Solanum lycopersicum* L., paradajz, Solanaceae	Fruit	E: furuncle	[18]
*Solanum americanum* Mill., pomoćnica, Solanaceae	/	E: oedema	[22]
	Leaf	E: leaf should be applied directly on inflamed skin areas	[23]
	Juice from the plant	E: reduces greasy dandruff. Also, it can be applied in the treatment of itching in diseases such as psoriasis and lichen	
	Fruit	E: for the extraction of pus from wounds on the leg or hand. Afterwards the wound was heated in an oven or using a candle in order to stop the progress of the infection	[29]
		E: applied on callus	[19]
*Solanum melongena* L., patlidžan, Solanaceae	/	E: against pustules on fingers. The eggplant should be halved, sprinkled with ammonium chloride, and both halves should be wrapped around the diseased finger, previously washed with brandy (“komovica”)	[4]
*Solanum tuberosum* L., krompir, Solanaceae	Root (tuber)	E: treatment of burns, and a disease called ‘rusa’	[18]
	/	E: against burns. Mixed with salt, it should be applied directly (in Herzegovina)	[5]
*Solanum dulcamara* L., trava ugaslica, Solanaceae	Leaf	E: Against oedema. Leaves should be crushed, mixed with brandy and applied on the affected area	[4]
*Sonchus oleraceus* (L.) L., zmijina trava, Solanaceae	/	I: against snakebites. The plant should be eaten, mixed with honey	[5]
*Stachys officinalis* (L.) Trev., ranilist, ranjenik, Lamiaceae	Aerial part	E: wounds	[18]
		E: snakebites (applied directly)	
		I: snakebites (juice from fresh plants)	
	/	E: wounds	[22]
		E: for gunshot wound. Crushed plant should be mixed with olive oil and applied to the wound	[5]
*Staphisagria macrosperma* Spach, [*Delphinium staphisagria* L.], trava od buva, Ranunculaceae	Aerial part	E: against scabies, nits	[23]
*Succisa pratensis* Moench., [*Asterocephalus succisa* Wallr.], preskočica, piskavac, Caprifoliaceae	Leaf	I: carbuncle (tea mixed with wine)	[18]
	Leaf, flower, and root	I: in the form of syrup or tea, it can be used for body detoxification in the elimination of eczema	[23]
*Symphytum officinale* L., [*Consolida major* Gilib.], gavez, Boraginaceae	Root	E: dog bite, baldness, wounds, and scabs	[18]
*Symphytum tuberosum* L. žuti gavez, trava od utrobe, Boraginaceae	/	E: dressing of shallow wounds	[5]
*Tanacetum balsamita* L., [*Balsamita major* Desf.], kaloper, Asteraceae	Aerial part	E: “rusa” (balm, compress)	[18]
*Teucrium chamaedrys* L., podubica, Lamiaceae	Aerial part	E: wound (herbal wine applied directly for wound cleaning)	[35]
*Teucrium polium* L., trava pepeljuša, Lamiaceae	Whole plant	E: oedema. The whole plant should be boiled, crushed and fried in oil. Subsequently it should be applied lukewarm as a poultice	[24]
*Thymus serpyllum* L., majkina dušica, Lamiaceae		E: tinea (mixed with warm water for compress)	[18]
	Whole plant	E: against lichen. The plant should be boiled and applied directly to the infected area. Additionally, the skin should be washed with water in which the plant was boiled	[17]
*Thuja occidentalis* L., tuja, Cupressaceae	Leaf	E: compression should be prepared with leaf decoction and applied directly on anal warts	[23]
*Tilia cordata* Mill., lipa, Malvaceae	Wood	E: wounds (pulverised charcoal made from linden wood applied directly)	[18]
	/	E: burns. A cloth soaked in brandy (‘rakia’) should be attached to the affected skin, and when the burn blister breaks, it should be covered with pulverised charcoal made of linden wood	[22]
	Wood	E: against itching. A bath should be made from linden wood, in which patient should bath three consecutive mornings. Bath should not be made from linden bark	[24]
	Leaf	E: for larger burns. Linden leaves cooked in milk should be applied as a poultice	[24]
	/	E: treatment of smelly feet. They should be sprinkled with crushed linden charcoal	
	/	E: treatment of ‘live’ wounds. Bleeding of wounds should be induced with very salty lye and afterwards sprinkled with finely crushed and sifted linden charcoal	[4]
*Trifolium pratense* L., [*T. purpureum* Gilib.], kravljača, detelina, Fabaceae	Flower	E: snakebites	[18]
*Trigonella foenum-graecum* L., piskavica, pjeskavica, Fabaceae	Seed	E: furuncle, oedema, open wounds, burns (cataplasm)	[35]
*Triticum aestivum* L., pšenica, Poaceae	Seed	E: bruises (balm)	[18]
		E: against ulcers. Flour, well sifted, mixed with white egg, tar and honey and this mixture should be placed directly on the ulcer to stay during day and night, after that it should be replaced with fresh balm	[4]
		E: against feet sweating. Fine flour should be baked in hot lard, then cooled and crushed into a powder that should be further used for sprinkling on feet	[4]
	/	E: against erysipelas (when it affects the skin on the face). Sourdough should be made from wheat flour and the whole head should be covered with it, except the nostrils. That dough should stand on the face for seven days	[28]
	Seed	E: against insect bites. A dish called “mandarica” should be made from fried flour, in which a small amount of water should be added until a thick porridge is prepared. It should be split on a cloth and applied to a bitten place	[28]
*Tropaeolum majus* L., [*T. elatum* Salisb.], dragoljub, Tropaeolaceae	Leaf and fresh seeds	E: prevents hair loss (in combination with fresh nettle leaves and common box leaves—a tincture is prepared which should be rubbed in)	[23]
*Tussilago farfara* L., podbel, Asteraceae	Leaf	E: wounds, sting	[18]
		E: bruises, cuts (after washing the leaf covered with fat is being placed on the injured area)	[35]
	Leaf	E: for wounds and pus cleaning. Directly applied	[21]
	Leaf	E: against wounds. Bandage regularly	[4]
	Leaf	I: scrofula (boiled leaf or juice from pressed fresh leaves)	[23]
	Flower, leaf	E: acne (warm compress)	
		E: applied on wounds with *Plantago lanceolata*	[20]
*Ulmus glabra* Huds., [*U. campestris* L.], brest, Ulmaceae	Bark	E: bruises (boiled in cow’s milk and applied as poultice)	[18]
	Root	E: for burns affecting large area. After removing root bark, it should be scraped off and applied on skin	[24]
*Urtica dioica* L., kopriva, Urticaceae	Leaf	E: bites (by snake and dog), wounds, bruises, hair loss	[18]
	/	E: skin ulcers (called nicina). Stinging nettle should be finely chopped and, together with brandy (“rakia”) used for dabbing the affected area, which furthermore should be wrapped	[28]
	Whole plant	E: hair should be washed with water in which plant was boiled to make hair grow better	[19]
	Leaf	E: against eczema. Leaf should be crushed and cooked with oil. After cooling, this mixture should be applied directly on eczema	[32]
	/	E: cuts. Fresh plant should be rubbed directly on the cut	[21]
	Root	E: against chronic (‘live’) wounds. Crushed root should be fried in fat and applied to the wound	[22]
	Root	E: against contusions. Root should be bruised with common salt and applied directly	[5]
	Aerial part	E: against oedema. Boiled stinging nettle should be placed on oedema. Instead of nettles, green plum leaves can also be used	[24]
*Valeriana officinalis* L., odoljen, Caprofoliaceae	Root	E: hair growth stimulator	[18]
*Veratrum album* L., čemerika, Liliaceae	Root	E: wounds	[40]
*Verbascum densiflorum* Bertol., divizima, Scrophulariaceae	Flower	E: it is used in combination with “blistering” balm for the treatment of chronic (“live”) wounds. Balm is used for wounds, opening, and cleaning. When it opens, it should be spread with *V. densiflorum* pulverised flower, or this pulvis can be mixed with laurel oil and applied directly “Blistering” balm can be made like this: inner bark from black elder branches, washed and dried root of yarrow, white olibanum, and spruce resin should be cooked in olive oil. After squeezing and cooling, a balm is obtained.	[5]
*Veronica beccabunga* L., razgon, Plantaginaceae	/	E: against oedema	[22]
*Veronica chamaedrys* L., zmijina trava, Plantaginaceae	/	E: snakebite	[22]
*Veronica officinalis* L., razgon, Plantaginaceae	Aerial parts	E: wounds	[18]
*Verbena officinalis* L., ljutovnica, Verbenaceae	Aerial part	E: wounds	[18]
*Veronica teucrium* L., zmijina trava, Plantaginaceae	/	E: snakebite	[22]
*Vicia faba* L., bob, Fabaceae	Seed	E: snakebite (roasted seed placed on the bite site)	[28]
*Viola odorata* L., ljubičica, Violaceae	Leaf	E: hair loss prevention (hair rinsing with water in which leaves of violet, cherry, sour cherry, and strawberry were boiled)	[18]
	Aerial part	E: against hair loss (rinse hair with tea prepared from violet)	[37]
*Viola tricolor* L., dan i noć, Violaceae	Flower	I: eczema, psoriasis, “rusa”	[23]
*Vitex agnus castus* L., konopljika, Lamiaceae	Aerial parts, fruit	E: wounds	[35]
*Vitis vinifera* L., vinova loza, Vitaceae	Stem	E: hair growth stimulator (juice produced by the vine during pruning in spring is used by women for hair treatment)	[18]
	/	E: against hair loss and dandruff. Wood ash obtained from burned grape vine should be mixed with hot brandy (“rakia”) and a piece of soda. This mixture should be applied to the hair every second day during the period of 40 days	[4]
	Juice	E: to prevent hair loss and stimulate hair growth. Juice that comes from grape vines when they are pruned in the spring should be smeared directly on the hair	[19]
*Zea mays* L., kukuruz, Poaceae	Seed	E: burns (poultice made from corn flour)	[18]
	Seed	E: against crusts. Corn seeds should be placed on a flat surface and pressed with hot tongs until the oil leaks out. This oil should be smeared on the crusts	[21]
	Seed	E: for minor burns. A porridge made from corn flour and wine should be applied directly on the wound	[24]
	Corncob	E: against crusts. A two-year-old corncob and a piece of discarded traditional leather shoes (“opanak”) should be burned, mixed, and beaten into a fine powder. The crusts must be washed first with clean soap, then dried, smeared with oil, and salted with the obtained black powder. This should be done for seven days, in the morning and in the evening	[4]
	Seed	E: against burns. Corn flour dough should be moistened with hot “komovica” and salted. Salt should also be applied on the burn, which should be subsequently covered with the obtained dough	[4]
		Corn flour dough should be well moistened with strong wine vinegar and applied as a poultice to the wound (ibid).	
	/	E: against erysipelas. The affected area should be covered with dough prepared from corn flour and hot brandy (“rakia”).	[28]
	/	E: against burns. The wound should be treated with a poultice made from corn flour and brandy (“rakia”) until it gets white. Subsequently, the wound should be smeared with human urine.	[21]
	Seed	E: against burns. Poultice should be made from corn dough prepared with brandy (“rakia”).	[28]

The skin problems identified in our study can be categorized into three main groups: hair and scalp issues, bites, and various skin conditions (primarily inflammatory), such as eczema and psoriasis. Among these, treatments and plants for wound healing were the most prominent.

### 2.1. Treatment of Hair and Scalp Problems

Hair has always been a symbol of beauty. It is also a motif that features prominently in Serbian oral poetry. Long hair arranged in a braid, or a pigtail, used to “enchant maidens” not only as a sign of male beauty, but of masculine power as well [6]. Historical sources from the early 19th century reveal that in the aftermath of the First Serbian Uprising, the Grand Leader Karađorđe, seeing how the Turks used to grab Serbian warriors by the hair and overpower them in battle, subsequently ordered all Serbian fighters to remove their pigtails. However, this order did not meet with popular approval in liberated Serbia, as many men bluntly disobeyed Karađorđe’s demand and wept over their cut-off braids when forced to submit to higher authorities.

Although hair problems are not life-threatening, they can have a significant impact on a person’s self-esteem and, consequently, their quality of life. According to our findings, in 19th- and 20th-century Serbia, the main hair-related issues were balding, hair loss, dandruff, head lice, nits, and scalp crusts. We identified 27 plant species from 21 different plant families that were used to manage these problems. The most representative families were Asteraceae, Ranunculaceae, Caprifoliaceae, Rosaceae, and Brassicaceae. The recorded plant species were mainly applied topically, either directly or in the form of various herbal preparations that were rubbed onto the scalp. These preparations primarily consisted of water in which the plant was soaked or boiled, as well as juice extracted from fresh plant parts (such as from the stem of *Vitis vinifera* and the fruit of *Solanum americanum*). Plants were mostly used individually, but combinations of plants were also recorded. For example, to prevent hair loss, hair was rinsed with water in which the leaves of *Viola odorata* (violet), *Prunus avium* (cherry), *Prunus cerasus* (sour cherry), and *Fragaira vesca* (strawberry) had been boiled.

While the exact ways in which most plant species promote hair growth or prevent hair loss are still not fully understood, it is believed that their effects may be due to factors like improved blood circulation, stimulation of the anagen dermal papillae, inhibition of dihydrotestosterone, anti-inflammatory properties, and better nourishment [41]. Previous studies have demonstrated the effectiveness of some species listed as being used for hair problems in our research.

A recent study showed that *Polygonatum multiflorum* extract promoted hair growth in telogenic C57BL6/N mice by triggering the anagen phase. When applied both topically and orally, it increased the length and number of hair follicles by upregulating the expressions of β-catenin and sonic hedgehog proteins [42]. Several studies have reported the anti-androgenic effects of *P. multiflorum* extract in prostate cancer cells, achieved through the suppression of 5-α reductase, an enzyme crucial for the production of dihydrotestosterone (DHT). Elevated levels of DHT can negatively affect hair follicles and shorten the hair growth cycle, leading to hair loss. The compounds 2,3,5,4′-tetrahydroxystilbene-2-*O*-β-D-glucoside (TSG) and emodin, found in *P. multiflorum* extract, are primarily responsible for its hair growth-promoting properties [43].

*Allium sativum* (garlic) has also attracted attention in clinical research for the treatment of alopecia areata. In a double-blind, randomized controlled trial involving participants with alopecia areata, a gel containing garlic extract as the active ingredient significantly enhanced the therapeutic effects of topically applied betamethasone. This suggests that garlic extract may be an effective adjunctive therapy for this condition [44]. The exact mechanisms by which garlic stimulates hair growth are still unknown. However, many researchers have shown that alopecia areata is an autoimmune condition that attacks hair follicles, leading to hair loss. Garlic’s immune-modulating effects may help explain its effectiveness in treating alopecia areata [45].

Previous studies have also confirmed the hair-promoting activity of compounds isolated from *Capsicum annuum* extracts. Namely, Harada et al. demonstrated that capsaicin may enhance the production of insulin-like growth factor-I (IGF-1) in the hair follicles of both mice and humans with alopecia [46]. This hormone plays a role in regulating hair growth by modulating cellular proliferation and migration during the development of hair follicles [47]. The effects of capsaicin on hair growth may result from the activation of skin sensory neurons. Specifically, capsaicin activates the vanilloid receptor-1, which in turn promotes the release of calcitonin gene-related peptide (CGRP) from sensory neurons, ultimately leading to an increase in IGF-I production [46].

Takahashi et al. demonstrated that proanthocyanidins from *Vitis vinifera* (grape) seeds [48], which are also present in stem winemaking byproducts [49], promote hair follicle cell proliferation in mice by 230% compared to the control (100%). According to the authors, these proanthocyanidins exhibited significant activity in converting the hair cycle from the telogen to the anagen phase in in vivo test systems using C3H mice.

*Urtica dioica* is already available in various commercial forms and has demonstrated anti-androgenic effects, acting as a 5-α reductase inhibitor [50]. The primary compounds responsible for these effects include phenolics, fatty acids, phytosterols, and lignins [51].

Nevertheless, some plant species were mentioned for the first time in relation to these indications, and due to their insufficient investigation, they represent significant potential for further studies: *Dipsacus fullonum*, *Inula helenium*, *Ligusticum officinale*, *Pteridium aquilinum*, *Raphanus raphanistrum*, and *Valeriana officinalis*. Additionally, there is no scientific data to confirm the use of *Juglans regia* leaves for hair loss, although this usage has been recorded in other regions of the world, such as northeastern Algeria [52]. In Nepal, however, a paste made from the bark of this plant species is used as a treatment for hair growth [53]. For some plant species, the use of specific plant organs has been recorded for the first time. For example, recent reports highlight the potential beneficial effects of fixed oil obtained from *Prunus avium* seeds [54]. In Serbia, during the 19th and early 20th centuries, the leaves of *P. avium* were used as part of a herbal preparation for hair loss prevention.

Dandruff is another dermatological condition limited to the scalp, characterized by itchy, flaking skin without visible inflammation [55]. The primary underlying pathogenic mechanisms involve *Malassezia* proliferation, along with local skin irritation and inflammation [56]. Conventional treatment typically includes the topical application of antifungal and anti-inflammatory agents. According to our study, *Allium cepa*, *Sinapis arvensis*, *Solanum americanum*, and *Vitis vinifera* were used for dandruff treatment in 19th and 20th-century Serbian folk medicine. Shams-Ghahfarokhi et al. highlighted that *A. cepa* extract could serve as a potential treatment for fungal infections [57], especially those caused by *Malassezia furfur*. Similarly, Simonetti et al. reported that *Vitis vinifera* seed extract effectively inhibited the growth of *Malassezia furfur*, with minimal inhibitory concentrations ranging from 32 to 161 mg/mL [58]. The authors observed that the antimicrobial activity was inversely related to the concentration of polymeric flavan-3-ols. During the 19th and early to mid-20th centuries, when families were larger and people lived in more densely populated settlements, head lice infestations (*Pediculus humanus* var. *capitis*) were more prevalent. *Actaea spicata*, *Consolida regalis*, and *Delphinium staphisagria* were traditionally used to treat lice infestations. These plants, belonging to the Ranunculaceae family, contain alkaloids known for their insecticidal properties [59]. Similar uses have been reported in Turkey, where *Consolida* and *Delphinium* species were employed in the treatment of body lice [60]. Plants from these genera have also been employed as insecticides for controlling various agricultural pests. Methyllycaconitine, a compound found in *Consolida* species, has been shown to bind strongly to the nicotinic receptors in insects [61]. Wang et al. highlighted diterpenoid alkaloids from *Delphinium* species as a promising candidate for developing new insecticides [62].

### 2.2. Treatment of Bites

Our study found that 32 plant species from 19 families (mainly Amaryllidaceae, Asteraceae, Solanaceae, Lamiaceae, Fabaceae, and Plantaginaceae) were used in the treatment of snakebites, as well as bites from bees, wasps, hornets, dogs, green lizards, and scorpions. Snakebites, in particular, posed a significant threat, especially to agricultural workers, due to limited access to healthcare and the lack of antivenoms. This often made them life-threatening or led to severe local injuries and complications. According to our findings, during the 19th and early to mid-20th centuries in Serbia, external applications were preferred for treating bites. The methods for preparing these remedies were simple and practical. Fresh plants or their products (such as juice or latex) were directly applied, rubbed over the bites, or mixed with water or oil to create a paste. Additionally, plants such as *Allium cepa*, *Helianthus annuus, Sonchus oleraceus*, and *Stachys officinalis* were taken orally. Plant species used in the treatment of bites typically exhibit symptomatic effects or act as antidotes [63]. Symptomatic effects focus on alleviating or eliminating pain, edema, hemorrhage, and necrosis. In contrast, antidotes counteract venom action through mechanisms such as enzyme inhibition and protein precipitation, which are the most commonly recognized processes. It has been shown that phospholipases, which are components of venom, can induce a wide range of pharmacological effects, including neurotoxicity, myotoxicity, and edema-inducing activity. Following local myonecrosis, the ensuing inflammatory response is critical to the long-term damage observed, which may ultimately lead to the loss of limb function or even amputation [64].

According to Felix-Silva et al. [65], only a few species recorded to be used traditionally against snakebites were tested preclinically, utilizing different snake venoms. Among plant species that were used for treatment of snakebites in Serbia during the 19th and 20th centuries, only *A. cepa* bulbs showed promising phospholipase A2 (PLA2) inhibition [66]. Also, for rosmarinic acid, which is the predominant polyphenolic compound in *Ocimum basilicum* extracts [67], Ticli et al. showed that it inhibits myotoxic activity and PLA2 [68]. Isoquercitrin and quercitrin, dominantly present in *Sambucus ebulus* [69], have been shown to inhibit hemorrhagic activity of different viperoid and elapid venoms, as well as some isolated snake venom metalloproteinases [70].

Besides snakebites, stings of the members of Hymenoptera, such as bees, hornets, and wasps, can also be fatal. They are commonly connected with symptoms such as erythema, itching, pain, and local swelling and subsequently can induce secondary bacterial infection [71]. Our study showed that only a few species were used in the treatment of insect bites: *Allium ampeloprasum, Ocimum basilicum, Prunus domestica*, *Satureja hortensis, Triticum aestivum*, and *Tussilago farfara*. Currently, there are no studies that can confirm their efficacy. They are most likely to offer symptomatic relief due to their anti-inflammatory, analgesic, or anti-pruritic effect. For example, due to the high content of tannins and anthocyanins in the fruit of *P. domestica*, the extract of this plant species exhibited high anti-inflammatory activity, antinociceptive efficacy in rats [72], as well as significant antibacterial efficacy [73].

### 2.3. Treatment of Various Skin Diseases

In this category, skin diseases were grouped into various types, including pimples, mumps, measles, wounds and cuts, boils, skin burns, abscesses, furuncles, ulcers, inflammation, psoriasis, eczema, acne, impetigo, lichen, contusions, scabies, anthrax, erysipelas, skin tuberculosis, warts, rashes, and pruritus.

According to the data collected in our study, the largest number of plant species was used for wound healing (54 species). Skin wounds occur when the skin structure is damaged as a result of different types of injuries, burns, cuts, or some pathologic conditions, resulting in damage to tissue, disruption of blood vessels, extravasations of fluids, and similar conditions, which can be found in diabetes or some vascular diseases. The physiological process of wound healing consists of several stages leading to wound recovery. They include hemostasis, inflammation, proliferation, and maturation, and are related to the contribution of different cells close to the wound site [74].

Skin wounds can be classified as acute or chronic. Acute injuries usually pass through a 2 to 4-week repair process when the epidermal structure and integrity are efficiently restored [75]. Chronic wounds usually manifest an incorrect healing process with a long time for cure and difficulties in anatomical and functional integrity. In that type of wound, the inflammatory phase is prolonged and is characterized by high levels of reactive oxygen species (ROS), pro-inflammatory cytokines, the presence of myeloid cells, proteases, and a damaged structure of the extracellular matrix. Moreover, vascular defects lead to chronic wound hypoxia and ischemia. Another significant problem with chronic wounds is frequent colonization by bacteria such as *Pseudomonas aeruginosa* or *Staphylococcus aureus*. All these points point to the fact that treating complex wounds such as burns, diabetic wounds, wounds with chronic infections, or large wound defects is very demanding.

Herbs and herbal remedies have a long tradition in wound healing. Given the fact that inflammation, disturbed oxidative status, and immune response, bacterial infections are common in wounds, the preferred biological activities of wound healing herbs include anti-inflammatory, antimicrobial, and antioxidant. Moreover, effects such as promotion of fibroblast proliferation or enforced collagen deposition, collagen synthesis activation, angiogenesis activation, protein expression, and blood clotting are of importance.

Scientific data have justified wound healing properties for several plant species that, according to our study, were used in the 19th and 20th centuries for wound treatment.

*Achillea millefolium* has been, since ancient times, one of the most frequently used plant species. Due to its anti-inflammatory, antiseptic, antioxidant, and astringent activity, it is used for treating wounds, cuts and burns. Anti-inflammatory, tyrosinase, and antioxidant activity of yarrow are dominantly connected with flavonoid compounds present in aerial parts [76]. Hajhashemi et al. conducted a double-blind clinical trial with 140 primiparous women [77]. They studied the efficacy of yarrow ointments on episiotomy. Ecchymosis, edema, redness, discharge, wound dehiscence, and pain level as healing process indicators were assessed at the 7th, 10th, and 14th day of the study. Redness, edema, ecchymosis, and pain level were less or more effective compared to the control groups.

Root extract of *Arctium lappa* significantly improved dermal extracellular matrix (ECM) metabolism and influenced the Wnt/-catenin signalling pathway known to be a regulator of wound healing in canine dermal fibroblasts, by regulating gene expression and cell adhesion [78]. In a pilot study conducted among Amish patients, it was exhibited that commercial preparation Burns and Wounds™ topical ointment (B&W) with burdock leaf extract was more effective in pain reduction and healing of first- and second-degree burns compared to the control [79].

The most significant activities of *Calendula officinalis* include anti-inflammatory, antibacterial, antifungal, anti-edematous, immunostimulant, and antioxidant and are connected with the presence of flavonoids, phenolic acids, terpenoids, carotenoids, quinones, and coumarins. A number of studies have proved that *C. officinalis* possesses good wound healing properties. Preethi and Kuttan studied the effects of marigolds on rat excision wounds [80]. On the 8th day after wound formation, a 90.0% wound closure rate was noticed in the extract-treated group, while it was 51.1% in the control group. Moreover, the contents of hexosamine and hydroxyproline were significantly higher in the extract-treated group compared to the untreated group. It demonstrated stimulation of fibroblasts’ proliferation and migration, and upregulation of the expression of some growth factors such as TGFβ1, bFGF, and CTGF in studies in vitro, while in in vivo studies, it promoted collagen deposition, enhanced angiogenesis, and accelerated re-epithelization.

*Urtica dioica* is also known for its wound-healing properties. Bouassida et al. showed that hydroethanolic extract of nettles possesses hemostatic as well as wound healing activities [81]. Among active ingredients, lupeol is highlighted due to its ability to improve re-epithelialization in the wound healing process.

Previous studies have shown that the kinetics of wound epithelialisation and contraction were significantly increased by topical use of *Trigonella foenum-graecum* (fenugreek seed). Fenugreek is rich in steroidal saponins that can reduce inflammation. Sumitra et al. showed that wounds in rats epithelialized faster compared to control wounds when the rats were orally treated with fenugreek seed suspension [82]. They concluded that fenugreek seed can produce different activities in various phases of wound repair.

Confirmation of the positive effect of *Punica granatum* L. (pomegranate) peel extract on wound healing has also been scientifically proven. Zhang et al. showed significant positive effects of pomegranate peel extracts in the minipig second-degree burn model [83]. The effect was connected with increases in TGF-β1 and VEGF-A protein and levels of gene expression. Hydroalcoholic pomegranate peel extract-based ointment used in the study of Hayouni et al. considerably improved the time of epithelialization and wound contraction in excised wounded models over 10 days through enhanced contraction rate, tensile strength, and the raising of DNA, protein, and collagen synthesis [84].

Lukiswanto et al. [85], reported recovery of skin burn wounds in rats with pomegranate whole fruit extract. They showed that the extract can speed up the healing of second-degree burn wounds characterized by a low number of inflammatory cells, complete and mature epithelium, and high density of collagen with good organization. Moreover, pomegranate flower extract incorporated in cream hastened wound healing in rats.

Often, in traditional and modern medicine, herbal preparations for the treatment of wounds contain several herbal components with different effects, such as antimicrobial, anti-inflammatory, and epithelizing. Morin et al. conducted a study with Cicaderma ointment, which contained extract of *C. officinalis*, *H. perforatum*, and *A. millefolium*, plant species that have been used since the mid-20th century for the treatment of wounds [86]. They confirm that the use of Cicaderma ointment promotes re-epithelialization, modulates inflammation, and accelerates wound healing in a model of skin ulcer in mice.

## 3. Materials and Methods

During the data collection process, we reviewed key ethnographic literature, including monographs, articles, and field reports, which extensively addressed the study of folk medicine in the 19th and 20th centuries. The texts analyzed in this study were primarily authored in the early 20th century, often as chapters in monographs that provided comprehensive descriptions of folk life across various regions of Serbia, such as Timok, Homolje, Zvižd, Levač, Temnić, Crna Reka, and others. The authors of these monographs were not professional ethnologists, but rather priests, teachers, and physicians who were based in these regions and conducted fieldwork over several years. In many cases, pioneering ethnographic research in Serbia that we considered was conducted by trained local amateurs or semi-professionals who were linked by lineage, marriage, and/or profession (teachers, priests, physicians) to the local communities they studied. This allowed them to conduct thorough fieldwork (which could—and in some cases did—last for years, or even a decade), as well as to gain genuine trust from their informants. This is important because the transmission of traditional healing knowledge in the local communities was usually followed by the oath of secrecy, and their procedures were not easily revealed to a researcher who was perceived as a stranger in the local community [21,87].

The consulted sources encompasses key contributions to the study of Serbian folk medicine, ethnomedicine, and traditional phytotherapy, including works by Čajkanović [18], Radulović [19,24], Pantelić [20], Bušetić [21], Đorđević [22,30], Gostuški [23], Petrović [25,34], Nikolić [26], Dragić [27], Grbić [28], Kostić [29], Rajković [31], Vuksan [32,37], Katanić [33], Tucakov [35], and Antonijević [36], alongside additional ethnographic and ethnobotanical studies that documente symbolic uses of plants, and traditional healing systems. This study excludes rituals, spells, and magical practices associated with traditional healing, as the analysis of such customs, while enlightening, does not directly align with the paper’s main objectives.

In their fieldwork, these authors rely in some cases on the instructions for collecting ethnographic data written by pioneers of Serbian ethnology, such as Jovan Cvijić, Tihomir Đorđević, and Jovan Erdeljanović [88]; in some cases, they followed the instructions authored by Dr Vladan Đorđević (and printed in his book on Serbian folk medicine, published in 1872 by Letopis Matice Srpske); finally, in some cases, they followed their own organizational plans. In addition, we gathered ethnographic data from two lexicons specialized in ethnobotany, Pavle Sofrić Niševljanin’s “Main Plants in Serbian Folk Religion and Oral Poetry” [40], and “Dictionary of Serbian Folk Beliefs About Plants”, written by Veselin Čajkanović [18].

The criteria for the literature selection depended exclusively on the availability of the consulted texts. All bibliographic sources used in this study were obtained from public libraries and journal archives, either in print or online. Specific collections or individual works can be accessed through national or university libraries, as well as digitized archives where available. A number of works and articles cited here were recently reprinted in collections of ethnographic and anthro-pogeographic works linked to specific regions (Homolje, Zvižd, etc.), but some that did not enter the study were either lost as a consequence of the destruction of libraries and archives in the World Wars or were in some other way unavailable. We have otherwise intended to include all the relevant empirical material when it comes to folk remedies for skin and hair-related problems in Serbian ethnography.

We used the internationally recognized classification system, the International Classification of Primary Care, as a consistent framework for organizing and presenting our material. The collected data were categorized according to the ‘S’ (Skin) chapter of this classification [89]. This chapter is sufficiently broad to encompass not only various skin diseases but also conditions such as external wounds, skin burns, bites from animals and insects, hematomas, contusions, as well as issues like hair loss and other hair-related problems. The names of the plant families were reported following the guidelines of the Angiosperm Phylogeny Group [90]. The identification and standardization of vernacular plant names were carried out using the Botanical Dictionary written by Dragutin Simonović, which served as the primary reference for matching folk names with accepted botanical taxa [91].

Finally, an extensive search of the scientific databases Scopus and SciFinder was conducted to gather recent findings related to the phytochemistry and potential mechanisms of action of the plant species mentioned. The collected data were also compared with plant-based treatments outlined in contemporary ethnopharmacological studies conducted in Serbia. It should be noted that the available literature does not provide sufficient information on adverse effects, toxicity, or potential risks (such as irritation, photosensitivity, or interactions with other products) associated with the reported plant species and preparations, which represents a limitation of this study.

Part of this work was previously presented at the 70th Annual Meeting of the Society for Medicinal Plant and Natural Product Research (GA), Thessaloniki, Greece, 2022 [92].

## 4. Conclusions

This study documents a rich and diverse ethnobotanical heritage related to the treatment of skin ailments, identifying 164 plant species from 63 families, along with one mushroom species, used traditionally in the investigated area. Preparation methods varied, often incorporating organic and inorganic substances to enhance efficacy, revealing a sophisticated understanding of synergistic effects. Unique ethnomedical practices, such as specific harvesting rituals and the use of urine or gunpowder in formulations, further reflect the cultural depth and empirical knowledge embedded in historical healing traditions. These results not only preserve valuable traditional knowledge but also indicate promising directions for future pharmacological research, especially concerning the bioactive potential of underexplored taxa like those from the Solanaceae family in skin disease management.

## Data Availability

Research data are included in the manuscript.
